# Chondrogenic Potency Analyses of Donor-Matched Chondrocytes and Mesenchymal Stem Cells Derived from Bone Marrow, Infrapatellar Fat Pad, and Subcutaneous Fat

**DOI:** 10.1155/2016/6969726

**Published:** 2016-10-03

**Authors:** John Garcia, Claire Mennan, Helen S. McCarthy, Sally Roberts, James B. Richardson, Karina T. Wright

**Affiliations:** ISTM, Keele University, Robert Jones and Agnes Hunt Orthopaedic Hospital NHS Foundation Trust, Oswestry, Shropshire SY10 7AG, UK

## Abstract

Autologous chondrocyte implantation (ACI) is a cell-based therapy that has been used clinically for over 20 years to treat cartilage injuries more efficiently in order to negate or delay the need for joint replacement surgery. In this time, very little has changed in the ACI procedure, but now many centres are considering or using alternative cell sources for cartilage repair, in particular mesenchymal stem cells (MSCs). In this study, we have tested the chondrogenic potential of donor-matched MSCs derived from bone marrow (BM), infrapatellar fat pad (FP), and subcutaneous fat (SCF), compared to chondrocytes. We have confirmed that there is a chondrogenic potency hierarchy ranging across these cell types, with the most potent being chondrocytes, followed by FP-MSCs, BM-MSCs, and lastly SCF-MSCs. We have also examined gene expression and surface marker profiles in a predictive model to identify cells with enhanced chondrogenic potential. In doing so, we have shown that Sox-9, Alk-1, and Coll X expressions, as well as immunopositivity for CD49c and CD39, have predictive value for all of the cell types tested in indicating chondrogenic potency. The findings from this study have significant clinical implications for the refinement and development of novel cell-based cartilage repair strategies.

## 1. Introduction

Autologous chondrocyte implantation (ACI) for the treatment of focal chondral and/or osteochondral lesions has changed very little since its inception [1], but there remains scope for improvement. While we and others have reported a significant level of improved joint function and a reduction in pain following treatment with ACI [2–4], disadvantages such as cost, potential donor-site morbidity, and the quality of repair tissue formed remain. Although we have shown donor-site morbidity to be minimal [5], there is also the added risk of chondrocyte dedifferentiation during culture expansion [6, 7], the extent of which is likely to impact on the ability of the chondrocytes to redifferentiate upon implantation into the defect site.

Mesenchymal stem cells (MSCs) isolated from the bone marrow (BM-MSCs) have been used in several clinical trials as an alternative cell source for use in cell therapies to treat cartilage injuries and osteoarthritis [8–10]. The process of acquiring a sample of bone marrow, however, results in an additional, painful procedure for the patient. The infrapatellar fat pad (FP) is often routinely removed and disposed of as surgical waste during arthroscopy or open knee surgery and may provide an accessible alternative source of MSCs (FP-MSCs) with demonstrable chondrogenic capacity* in vitro* [11, 12]. Another accessible source of MSCs, although studied to a lesser extent for their chondrogenic propensity, is MSCs derived from subcutaneous fat (SCF-MSCs) [13, 14]. The ability to utilise these tissues for the treatment of cartilage injuries has the potential to improve the way we currently treat patients.

An important factor to consider when comparing and contrasting the properties of different cell types is the “donor impact” as donor demographics, such as age and gender, are factors which are known to affect cell proliferation and differentiation capacity [15–17]. The impact of donor is particularly critical for autologous treatment regimes and in deciding whether such a cell-based therapy represents the appropriate treatment option for an individual patient. Unravelling the impact of tissue and donor source and developing tools to predict the efficacy of cell-based treatments will likely result in the refinement of existing treatments and may provide valuable additional information for consideration during the decision making process of cost benefit versus clinical efficacy.

In this study, we have examined 4 different cell types (chondrocytes, BM-MSCs, FP-MSCs, and SCF-MSCs) and tested the chondrogenic potential of each population of cells. This study compares donor-matched cell types and was designed to establish the impact of tissue source and donor on chondrogenic differentiation capacity and to continue the process of establishing a marker panel indicative of chondrogenic potency and likely clinical success. Such marker(s) could be screened for and used in the selection of a particular cell type and/or subpopulation of cells with enhanced chondrogenic capability prior to treatment. We envisage that taken together this information could significantly improve the success of cell-based therapies for cartilage injuries and perhaps even lead to the development of novel individualised treatments for cartilage repair.

## 2. Materials and Methods

### 2.1. Patients

All samples were obtained after patients had provided written informed consent; favourable ethical approval was given by the National Research Ethics Service (11/NW/0875) and all experiments were performed in accordance with relevant guidelines and regulations. Donor-matched samples of cartilage, BM, FP, and SCF were obtained from 5 patients (2 males and 3 females, ages 67–81 years) undergoing total knee replacement (TKR) surgery (Table 1).

### 2.2. Isolation of Chondrocytes

Macroscopically normal articular cartilage was excised from the femoral condyles of patients undergoing TKR. Cartilage tissue was weighed, minced into small pieces with a sterile scalpel, and digested in collagenase type II (250 IU/mg dry weight, Worthington, New Jersey, USA) for 16 hours at 37°C. The resulting suspension was passed through a 40 *μ*m cell strainer and centrifuged (350 ×g for 10 minutes) to produce a cell pellet that was resuspended in Dulbecco's Modified Eagle's Medium/F-12 (DMEM/F-12) with 1% (v/v) penicillin/streptomycin (P/S) and 10% (v/v) foetal calf serum (FCS, all Life Technologies, Paisley, UK), hereafter referred to as complete culture medium, at a seeding density of 5 × 10^3^/cm^2^.

### 2.3. Isolation of MSCs from Bone Marrow

Bone marrow aspirates and bone chips were obtained from the tibial plateau of patients undergoing TKR. Bone marrow was first diluted with an equal volume of phosphate buffered saline (PBS, Life Technologies) then split between two 50 mL tubes, layered onto 10 mL of Lymphoprep*™* (Alere Technologies AS, Oslo, Norway), and centrifuged (900 ×g for 20 minutes). The buffy coat, containing mononuclear cells, was aspirated and added to complete culture medium and centrifuged (750 ×g for 10 minutes). The resulting cell pellet was resuspended in complete culture medium and BM-MSCs were seeded at a density of 20 million cells per 75 cm^2^ tissue culture flask. Cells were left to adhere for 24 hours before the media were changed and the nonadherent cells were removed. Bone chips were placed in a 175 cm^2^ culture flask with 30 mL of complete media for 7 days to allow the plastic adherent cells to migrate out of the bone chips.

### 2.4. Isolation of MSCs from Adipose Tissues

Human FP and SCF tissue samples were obtained from patients and processed within 2 hours of receipt from the operating theatre. The FP was dissected from the innermost zone, to avoid contamination with synovium derived cells as described previously [12]. Dissected FP and SCF tissues were washed in PBS, minced, and digested with 1 mg/mL collagenase type I (≥125 digesting units/mg, Sigma-Aldrich, Poole, UK) in serum-free media for 1 h at 37°C. The resulting cells were strained through a 40 *μ*m nylon cell strainer and centrifuged (350 ×g for 10 min). Cells were then seeded at a density of 5 × 10^3^/cm^2^ in complete culture medium. All cultures were maintained in a humidified incubator at 37°C and 5% CO_2_.

### 2.5. RNA Extraction and Quantitative Real-Time Polymerase Chain Reaction (qRT-PCR)

After trypsinisation at passages 3-4, 2 × 10^5^ monolayer cells were centrifuged (500 ×g for 5 minutes), frozen in liquid N_2_, and stored at −80°C briefly prior to extraction. Cells were thawed on ice and messenger RNA (mRNA) was extracted using an RNeasy® extraction kit (Qiagen, Hilden, Germany) according to the manufacturer's recommendations. RNA was converted to cDNA using a High-Capacity cDNA Reverse Transcriptase Kit® (Applied Biosystems, Warrington, UK) according to the manufacturer's instructions. qRT-PCR was used to evaluate the expression of specific genes, indicative of chondrogenic potency or hypertrophy [18]. RT-qPCR analysis was performed on the Quant Studio 3 Real-Time Quantitative PCR System (Applied Biosystems) using SYBR green QuantiTect primer assays for the chondrogenic genes Sox-9, collagen type II (Coll II), aggrecan (ACAN), frizzled-related protein (FRZB), and the following genes indicative of hypertrophy: activin receptor-like kinase 1 (Alk-1) and collagen type X (Coll X). Peptidylprolyl isomerase A (PPIA) and TATA-binding protein (TBP) were used as reference genes (Qiagen). The relative expression of each gene was determined using the comparative C_T_ method [19].

### 2.6. Flow Cytometry

Flow cytometry was used to assess the immunoprofile of chondrocytes and MSCs prior to chondrogenic differentiation. Cells at passages 3-4 were harvested, pelleted, and resuspended in 2% bovine serum albumin (BSA, Sigma-Aldrich) in PBS. FC receptors were blocked for 1 h at 4°C using 10% (v/v) human IgG (Grifols, Barcelona, Spain) in 2% (v/v) BSA in PBS (immunobuffer). The cells were then washed with immunobuffer and centrifuged (350 ×g for 8 minutes). Cells were stained for 30 minutes at 4°C with fluorochrome conjugated antibodies against cell surface markers indicative of MSC according to the International Society for Cellular Therapy (ISCT) [20]. Markers probed for were CD90-phycoerythrin (PE) (clone 5E10), CD105-allophycocyanin (APC) (clone 266), and CD73-brilliant violet 421 (BV421) (clone AD2), CD19-BV421 (clone HIB19), CD34-APC (clone 581), CD45-PE (clone HI30), HLA-DR-APC (clone TU36), and CD14-PerCP-Cy5.5 (clone M*ϕ*P9). CD markers which have been reported as putative chondrogenic potency markers [21–28] were also probed for; these included CD49c-PE (clone C3 II.1), CD166-BV421 (clone 3A6), CD39-APC (clone TU66), CD44-peridinin chlorophyll protein-cyanine 5.5 (PerCp-Cy5.5) (clone G44-26), and CD271-BV421 (clone C40-1457) (BD Biosciences). Appropriate isotype-matched IgG controls were used throughout (BD Biosciences). Data from at least 10,000 stained cells is presented which was analysed using a FACSCanto II flow cytometer (BD Biosciences) and BD FACSDiva v.7.0 software.

### 2.7. Chondrogenic Differentiation

The chondrogenic potential of all cultured cell populations was assessed at passage 4 using an established 3D pellet culture system [12, 29]. Briefly, 2 × 10^5^ cells were centrifuged to produce a cell pellet which was maintained in DMEM F12, FBS (10% v/v), P/S (1%) ITS (1%, v/v), ascorbic acid (0.1 mM) (Sigma-Aldrich), dexamethasone (10 nM), and transforming growth factor *β*-1 (TGF-*β*1, PeproTech, London, UK) (10 ng/mL). After 28 days, cell pellets were frozen in liquid nitrogen and stored at −80°C prior to use. In total, *n* = 6 pellets per donor were produced, *n* = 3 for glycosaminoglycan (GAG)/DNA analysis and *n* = 3 for histological analysis.

### 2.8. GAG/DNA Analysis of Chondrogenic Pellets

Pellets were digested using papain to release GAGs and DNA. A digestion buffer consisting of 50 mM sodium phosphate (BDH), 2 mM EDTA (Sigma-Aldrich), and 20 mM N-acetyl cysteine (BDH) was prepared and the pH adjusted to 6. Papain (Sigma-Aldrich) was added to the digestion buffer to reach a final concentration of 125 *μ*g/mL. Each chondrogenic pellet was digested using 200 *μ*L of papain digest solution and placed in a 60°C oven for 3 hours. The digest suspensions were mixed vigorously every 30 minutes by vortexing the tubes. Samples were then centrifuged at 1000 ×g for 5 mins, aliquoted, and stored at −20°C until further use.

The dimethylmethylene blue (DMMB) assay was used to quantitate GAGs [30, 31]. Standards were prepared by dissolving chondroitin sulphate (Sigma-Aldrich) from bovine trachea in PBS to create appropriate serial dilutions. Fifty microlitres of sample or standard was added in triplicate to a 96-well plate and 200 *μ*L of the DMMB staining solution was added to each well. The absorbance was immediately read at *A*_530 nm_ and *A*_590 nm_. A standard curve was plotted (*A*_530 nm_/*A*_590 nm_) − (*A*_530 nm blank_/*A*_590 nm blank_), from which the total GAG content in each sample was calculated using the equation of the curve.

The PicoGreen® fluorescence assay (Invitrogen) was used to quantitate the amount of double stranded DNA in solution and was conducted according to the manufacturer's instructions. Fluorescence was read on a plate reader configured to excitation = 480 nm and emission = 520 nm. The normalisation of GAG content in chondrogenic pellets was achieved by dividing the total GAG content of a given pellet by the DNA content of that same pellet.

### 2.9. Histological Analysis of Chondrogenic Pellets

Pellets were cryosectioned (7 *μ*m) onto poly-L-lysine coated slides (Cell Path, Newtown, UK) and stained for GAGs with toluidine blue (BDH) metachromatic stain for 30 seconds and then washed briefly in tap water. Slides were left to air dry before mounting in Pertex (Cell Path). Chondrogenic pellets were assessed following toluidine blue staining using a modified version of the Bern score [32]. In brief, cells pellets were assessed using the following criteria: uniformity and intensity of toluidine blue staining and distance between cells/amount of matrix produced and cell morphology. Each of these three categories was scored from 0 to 3.

### 2.10. Statistical Analysis

The Shapiro-Wilk normality test was used to assess the distribution of quantitative data. A one-way ANOVA with Bonferroni's multiple comparisons test was used to test for significant differences between cell types with regard to gene expression, immunoprofile, GAG quantitation, and histological scores for chondrogenic pellets. Pearson's correlation coefficients were determined for gene-gene expression analyses and chondrogenic assessments (GAG quantitation and histological analyses). Multilevel modelling was conducted to determine whether gene expression and cell surface marker positivity were predictors of chondrogenic outcome as measured by GAG content of the pellet and histological scoring. In these models, cell source, gene expression, and cell surface marker positivity were considered as fixed effects, while the donor was considered as a random effect. The donor effect was determined using Wald's tests. Graphs are shown as means ± standard deviation, with statistical significance considered at ^*∗*^ *p* < 0.05, ^*∗∗*^ *p* < 0.01, and ^*∗∗∗*^ *p* < 0.001. All statistical analyses were performed in GraphPad Prism version 6 (GraphPad Software, California, USA) and SPSS version 20 (IBM, New York, USA).

## 3. Results

### 3.1. Chondrogenic/Hypertrophic Gene Expression prior to Chondrogenic Differentiation

**Figure 1:**
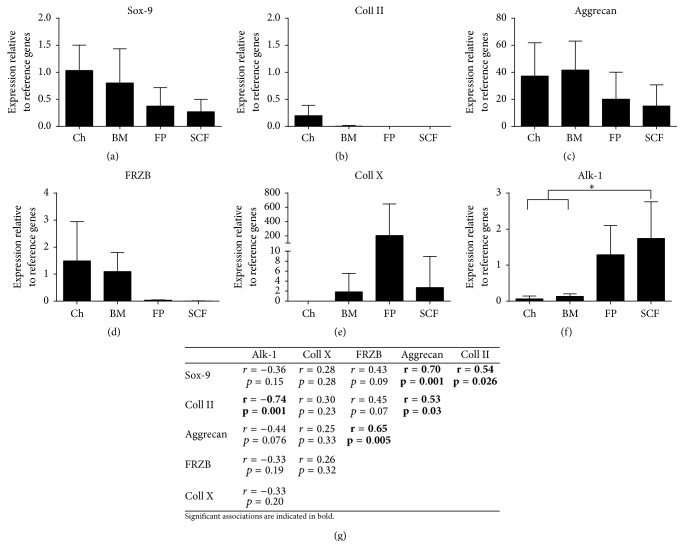
The expression of chondrogenic and hypertrophic genes in monolayer cell populations prior to chondrogenesis. ((a)–(f)) Chondrocytes (Ch), bone marrow MSC (BM), fat pad MSC (FP), and subcutaneous fat MSC (SCF). Data shown are the means ± the standard deviation of triplicate runs and 5 donors for each cell population. One-way ANOVA and* post hoc* Bonferroni tests were used to test for significant differences in gene expression levels between cell types. (g) Pearson's correlation analysis matrix comparing genes which may be predictive of chondrogenic potential; significant correlations are in bold. Gene expression is expressed relative to the reference genes PPIA and TBP.

Perhaps not too surprisingly, of the cell types tested in this donor-matched study, the chondrogenic potency genes (Sox-9, Coll II, aggrecan, and FRZB) were consistently expressed at the highest levels in culture expanded chondrocytes. Further, chondrocytes demonstrated the lowest expression profiles for the hypertrophic genes tested (Alk-1 and Coll X). Of the MSC populations that we have examined, BM-MSCs displayed chondrogenic and hypertrophic profiles that most closely resembled those of culture expanded chondrocytes. In contrast, the adipose sources of MSCs investigated (FP-MSCs and SCF-MSCs) were least like culture expanded chondrocytes and demonstrated the lowest chondrogenic potency and the highest hypertrophic gene expression profiles. SCF-MSCs expressed Alk-1 at significantly higher levels than chondrocytes and BM-MSCs (*p* = 0.044 and *p* = 0.034, resp.) (Figures 1(a)–1(f)).

Gene expression associations for all of the cell types examined in this study were tested using Pearson's correlation coefficient analyses and are presented in a correlation matrix (Figure 1(g)). Significant interactions noted between the chondrogenic potency genes were as follows: aggrecan was positively associated with Sox-9, Coll II, and FRZB; in addition, Sox-9 was positively associated with Coll II. There was also a significant negative association observed between Coll II and Alk-1 expression.

### 3.2. MSC/Chondrogenic Immunoprofiling

**Figure 2:**
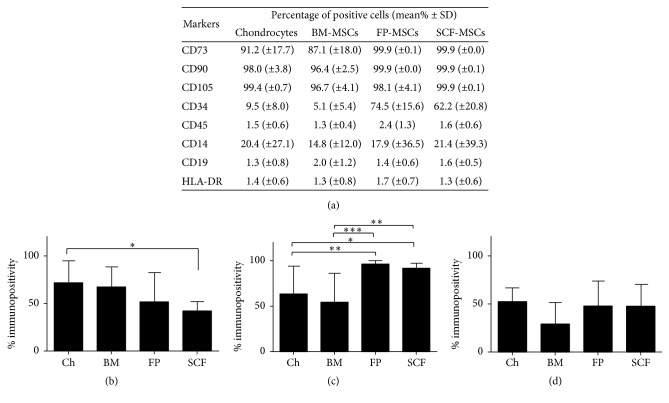
Immunoprofiles for MSC markers and putative chondrogenic potency markers of culture expanded cells prior to chondrogenesis. (a) ISCT MSC immunoprofiles. Immunoprofiles for the putative chondrogenic markers CD49c (b), CD166 (c), and CD39 (d). Flow cytometry was used to detect the percentage of positive cells for each marker on monolayer cell populations of chondrocytes (Ch), bone marrow MSC (BM), fat pad MSC (FP), and subcutaneous fat MSC (SCF) prior to chondrogenesis. Data shown are the means ± the standard deviation of 5 donors for each cell population. One-way ANOVA and* post hoc* Bonferroni tests were used to test for significant differences in the positivity of cell surface markers between cell types.

Flow cytometry analyses revealed immunopositivity for the MSC markers CD73, CD90, and CD105 for all of the populations of cells examined, but to varying levels. FP and SCF derived MSCs adhered to ISCT criteria (i.e., >95% positive); chondrocytes and BM-MSCs also adhered to ISCT criteria for CD90 and CD105 positivity but were <95% positive for CD73. All of the cell populations tested were <2% positive for CD19, CD45, and HLA-DR, in line with ISCT criteria. Some positivity was recorded in all of the cell populations tested for CD34 with high levels (62.2–74.4% positivity) seen in the adipose derived MSCs, which also adheres to ISCT [33]; in addition, >2% of BM-MSCs were also CD34 positive, which does not conform to recommendations by the ISCT [20]. CD14 was present on all cell populations, ranging on average between 14.8 and 21.4% positivity for each cell type (Figure 2(a)). Differences between cell types for putative chondrogenic potency marker positivity were noted for CD49c, CD166, and CD39 (Figures 2(b)–2(d)). Chondrocytes showed significantly greater positivity for CD49c compared to SCF-MSCs (*p* = 0.014), whereas the adipose derived MSCs showed significantly higher positivity for CD166 compared to chondrocytes or BM-MSCs (*p* = 0.0046 and *p* = 0.0002, resp., for FP-MSCs and *p* = 0.021 and *p* = 0.01, resp., for SCF-MSCs). No differences were noted for CD44 or CD271, in that all cell types were >95% positive for CD44 and <5% positive for CD271 (data not shown).

### 3.3. Chondrogenic Differentiation

**Figure 3:**
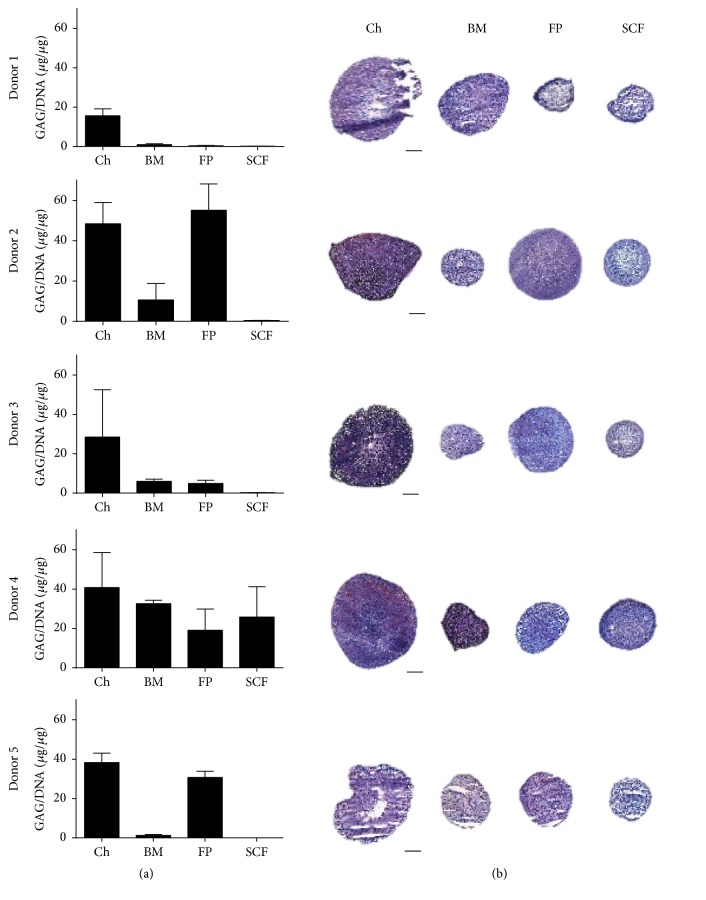
Chondrogenic assessments of pellet cultures between donors. (a) Production of GAG/DNA in pellet cultures from chondrocytes (Ch), bone marrow MSC (BM), fat pad MSC (FP), and subcutaneous fat MSC (SCF). GAGs were measured after chondrogenic differentiation using the DMMB assay and normalised to the DNA content of pellets; each donor is represented in individual graphs. Data shown are the means ± the standard deviation of triplicate pellets. (b) Chondrogenic pellets from Ch, BM, FP, and SCF showing representative toluidine blue staining for each donor. Scale bars represent 200 *μ*m.

**Figure 4:**
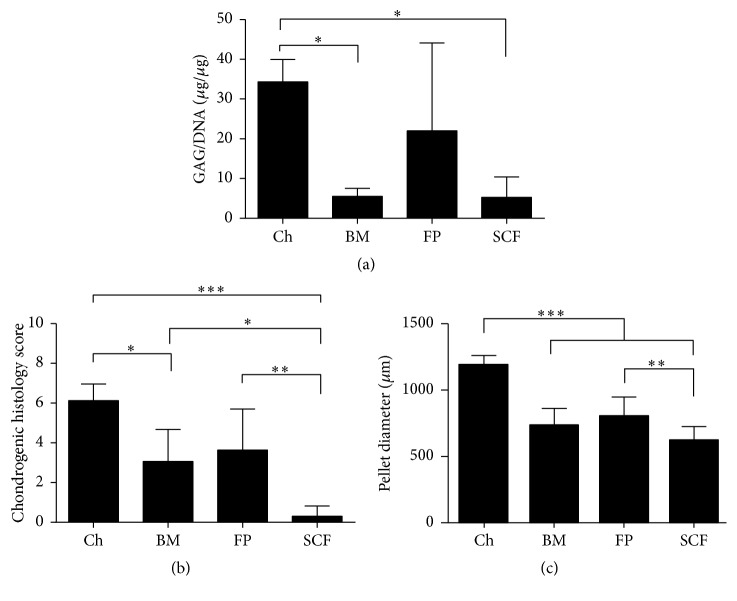
Chondrogenic assessments of pellet cultures across cell types. (a) Production of GAG/DNA in pellet cultures from chondrocytes (Ch), bone marrow MSC (BM), fat pad MSC (FP), and subcutaneous fat MSC (SCF) for all donors combined. (b) Chondrogenic histology scores for Ch, BM, FP, and SCF for all donors combined. (c) Mean chondrogenic pellet diameter (*μ*m) for Ch, BM, FP, and SCF for all donors combined. Data shown are the means ± the standard deviation of triplicate pellets and 5 donors for each cell population. One-way ANOVA and* post hoc* Bonferroni tests were used to test for significant differences between cell types.

After 28 days of differentiation, donors demonstrated variability in chondrogenic capacity across the cell types tested. When results from individual donors were examined, chondrocytes consistently produced chondrogenic pellets in terms of GAG quantitation and histological analyses, but the propensity for MSCs to undergo chondrogenic differentiation was variable between individuals. GAG/DNA analyses appeared to match the histological findings noted for each patient: that is, larger pellets with prominent matrix metachromasia had the highest levels of GAGs measured (Figure 3). Pearson's correlation analyses across donors confirmed that there was a significant association between pellet GAG quantitation and histological score (*p* = 0.01). When donors were grouped and chondrogenic analyses were performed comparing differentiation between cell types, chondrocytes consistently demonstrated the most pronounced chondrogenic differentiation in terms of GAG/DNA analyses; they produced significantly more GAG than BM and SCF derived MSCs (*p* = 0.032 and *p* = 0.030, resp., Figure 4(a)). In terms of histological quantitation and chondrogenic score SCF-MSC scores were significantly lower than chondrocytes, BM, and FP derived MSCs (*p* < 0.0001, *p* = 0.0195, and *p* = 0.0082, resp.) and chondrocytes scored significantly higher than BM-MSCs (*p* = 0.013, Figure 4(b)). Chondrocytes also produced the largest pellets (in terms of diameter) compared to any of the MSCs tested (*p* < 0.0001) and FP-MSC pellets were significantly larger than SCF-MSC pellets (*p* = 0.008, Figure 4(c)).

### 3.4. Chondrogenic Potency Analyses

Multilevel modelling analysis was performed in an effort to identify chondrogenic potency predictors prior to chondrogenic differentiation. This analysis demonstrated that Alk-1 expression and CD166 immunopositivity both negatively associated with GAG quantitation in pellet cultures. However, CD49c and CD39 expression positively associated with GAG quantitation and histological score, respectively. Sox-9 expression positively associated with chondrogenic histological score, whereas expressions of the hypertrophic genes Coll X and Alk-1 were shown to negatively associate (Table 2).

Further multilevel model analysis showed that cell type (but not donor source) had a significant impact on the predictive aspect of gene expression for both of the chondrogenic assessments tested, that is, GAG quantitation (*p* < 0.001) and histological outcome (*p* < 0.001). Similarly, cell type (but not donor source) had a significant impact on the predictive aspect of cell surface marker positivity with regard to both GAG quantitation (*p* < 0.001) and histological outcome (*p* < 0.001). Using an interaction term in a multilevel model consisting only of the cell types and the variable in question, results showed that the relationship between Sox-9/Alk-1 expression and the histological outcome varied significantly across cell source (*p* = 0.03 and *p* = 0.034, resp.), which was not the case for Coll X (*p* = 0.07). The relationship between Alk-1 expression and GAG quantitation also did not vary across cell types (*p* = 0.43). In terms of immunopositivity, the relationship between CD39 positive cells and histological outcome varied significantly between cell types (*p* < 0.001) as did the relationship between CD49c or CD166 positive cells and GAG quantitation (*p* < 0.001 for both markers).

## 4. Discussion

In recent years MSCs isolated from BM, FP, and SCF have been compared and contrasted extensively in* in vitro *studies [12, 13, 34–38] as alternative cell sources to the chondrocytes used in ACI. However, to our knowledge there are no studies to date that have compared the* in vitro* chondrogenic potency of these cell types together and from matched samples. In the present study, we have tested known gene profiles and immunoprofiles indicative of chondrogenic potential in a predictive model comparing chondrocytes, BM, FP, and SCF derived MSCs from 5 matched human donors. We have determined the expression of the chondrogenic genes, Sox-9, Coll II, ACAN, and FRZB, and the hypertrophy associated genes, Coll X and Alk-1, in these cell populations prior to chondrogenesis. In doing so, we have confirmed that across all of the cell populations tested, the master regulator of chondrogenesis, Sox-9, correlates with the expression of Coll II and ACAN and that the hypertrophy associated marker Alk-1 is negatively associated with Coll II expression. In addition, we have shown that there is a positive association between the expression of FRZB and ACAN, which has been previously reported in the chondrogenic ATDC5 cell line [39]. In terms of immunoprofile, the putative chondrogenic markers CD44, CD105, and CD271 were excluded from predictive analyses due to their uniform expression levels across cell types. CD49c, CD166, and CD39 were present on all of the cell populations examined to varying degrees and as such were taken forward into our multilevel chondrogenic potency analysis.

Following an established* in vitro *cell pellet chondrogenic differentiation procedure [12, 29] our quantitative assessment of GAG synthesis demonstrated that chondrocytes generate significantly more GAGs compared to BM-MSCs and SCF-MSCs but not FP-MSCs, with some notable variation between donors. Our findings are comparable to previous studies which have shown that chondrocytes and FP-MSCs display similar levels of GAG production and that FP-MSCs produce more GAGs than BM-MSCs [37] and donor-matched SCF-MSCs [38]. Further, our assessments for chondrogenic differentiation status in terms of GAG quantitation and histological score were significantly correlated. Chondrocyte-generated pellets consistently produced the highest scores, which were significantly greater than those formed by BM or SCF cell populations, whereas SCF pellets produced the lowest scores of all the cell populations examined. By simply measuring the diameter of pellets we have also shown that chondrocytes produced the largest pellets compared to BM and SCF derived pellets. Taken together, our chondrogenic assessments suggest that, not too surprisingly, culture expanded chondrocytes have the greatest propensity for chondrogenic differentiation* in vitro*, closely followed by FP-MSCs and then BM-MSCs, whereas SCF-MSCs consistently produced the worst chondrogenic outcome measures, regardless of the assessment used. These findings are corroborated by other studies that have reported paired comparisons between these cell types [12, 13, 34–38]. However, this is the first time, to our knowledge, that all four of these cell populations have been examined in the same study, allowing for a hierarchical chondrogenic potency comparison with the impact of donor taken into account by donor matching the samples tested.

The multilevel modelling analyses performed in this study have allowed us to explore the relationships between putative chondrogenic potency markers (gene expression and surface marker profiles) and chondrogenic outcome based on combined data from each cell source tested, while simultaneously examining the potential influence of donor and cell type. However, we have not yet verified whether the predictive factors for chondrogenesis that we have identified for combined data are present if only individual cell types are analysed as we believe that the small donor size precludes this type of analysis in the present study. We should of course be cautious when interpreting any analyses derived from a small donor sample size and we acknowledge this as a limitation of the study. Nonetheless, our multilevel modelling has revealed that the expressions of Alk-1 and Coll X are negatively associated with chondrogenic potential in terms of histology scores and for Alk-1 expression, as well as the GAG content of chondrogenically induced pellet cultures. In contrast, Sox-9 expression prior to chondrogenesis positively correlated with histological pellet scores. Some of these gene associations match a previous report comparing FP-MSCs and SCF-MSCs [38]. The novelty of our study is that some of these chondrogenic potency gene associations hold true across all of the cell types examined in the present study. The poor correlation between the baseline (predifferentiation) expressions of the chondrogenic genes Coll II and ACAN is noteworthy and comparable to findings in other studies [40, 41]. For example, Stenberg et al. (2014) have confirmed that the transplanted chondrocyte expression levels of ACAN and Coll II in ACI had no bearing on clinical outcome [40]. Further, Cote et al. (2016) demonstrated that the genome-wide transcription profile of Coll II and ACAN (amongst other genes) did not correlate with the production of GAGs in a single cell analysis of bovine MSCs and chondrocytes. The authors attribute this finding to the heterogeneity in single cell transcriptional profiles. It is extremely likely that the gene analysis in the present study derived from heterogeneous cell populations and four very different tissue sources will vary to an even greater extent.

In addition, our multilevel analysis indicates that CD49c and CD39 immunopositivity positively predicts GAG production and histological score, respectively, in cell pellets, with no significant difference observed between donors. Other studies have shown that CD49c positivity on chondrocytes and CD39 positivity on synovium derived MSCs are associated with increased* in vitro* chondrogenic potential [21, 27]; however, our results are the first to demonstrate these relationships across matched chondrocytes, BM-MSCs, and adipose derived MSCs. Perhaps surprisingly CD166 positivity did not indicate chondrogenic potential, as has been previously shown [24, 26]; in fact immunopositivity for this marker was negatively associated with chondrogenic assessments. One potential explanation for this finding might be that CD166 was expressed at significantly greater levels on SCF-MSCs compared to chondrocytes and BM-MSCs and that in our hands SCF-MSCs have been shown to consistently demonstrate a poor propensity for chondrogenic differentiation. Interestingly, we have demonstrated through this multicell type, donor-matched study that the source of cells significantly influences both GAG production in pellet culture and also the histological score of the pellet. In contrast, the donor had no demonstrable impact on either of the chondrogenic assessments tested, although as stated previously we must be cautious with this finding as our results are based on a small cohort of donors. Follow-up studies should be geared towards understanding the molecular mechanisms that account for the differences observed between cell populations and in the development of methods to select cells with enhanced chondrogenic potential.

## 5. Conclusions

We have demonstrated the chondrogenic predictive value of high levels of Sox-9 and low levels of collagen type X or Alk-1 expression as well as immunopositivity for CD49c and CD39 in a combined data analysis of chondrocytes, BM-MSCs, FP-MSCs, and SCF-MSCs. Further individual analyses on larger donor cohorts will be required to validate these findings for individual cell types before these predictive factors could be used as selection criteria prior to the transplantation or banking of each cell type in the treatment of cartilage injuries. We have also shown, using donor-matched samples, that cell type significantly influences the chondrogenic potency of the MSC sources examined in this study; we have demonstrated that MSCs sourced from the infrapatellar fat pad of the knee or bone marrow provide the “next best” alternative to chondrocytes, in terms of* in vitro* chondrogenic differentiation capacity. Further, our results have consistently shown that SCF derived MSCs have the poorest propensity for chondrogenic differentiation. These findings have important clinical implications, not only for the understanding of MSC chondrogenic differentiation capacity, but also for the development of cell therapy strategies to screen for and select potent cell types prior to application in the treatment of cartilage injuries.

## Figures and Tables

**Table 1:** Donor demographics.

ID	Gender	Age (years)	Pathology
Donor 1	Male	71	OA with extensive joint degeneration
Donor 2	Female	67	OA with loss of joint space
Donor 3	Female	75	Patellofemoral OA and loss of joint space in medial compartment
Donor 4	Female	81	OA
Donor 5	Male	74	OA with joint stiffness

**Table 2:** Multilevel modelling.

	GAG/DNA			Chondrogenic histology score		
	Coefficient	95% CI	*p* values	Coefficient	SE (95% CI)	*p* values
Sox-9	−5.6	−11.7, 0.5	0.07	**1.01**	**0.18, 1.84**	**0.02**
Coll II	22.3	−7.8, 52.4	0.14	3.96	−0.16, 8.08	0.06
Aggrecan	−0.003	−0.2, 0.2	0.97	−0.019	−0.04, 0.004	0.10
FRZB	33.1	−27.4, 93.6	0.3	0.86	−7.46, 9.18	0.83
Coll X	−0.01	−0.02, 0.003	0.12	**−0.002**	**−0.004, −0.0002**	**0.03**
Alk-1	**−9.5**	**−12.2, 6.7**	**<0.001**	**−0.61**	**−0.99, −0.23**	**0.003**
CD49c	**0.2**	**−0.1, 0.5**	**0.018**	−0.04	−0.02, −0.01	0.63
CD166	**−0.3**	**−0.5, −0.04**	**0.03**	0.01	−0.04, 0.034	0.16
CD39	−0.02	0.01, 0.4	0.78	**0.024**	**0.01, 0.04**	**0.002**

Significant associations are indicated in bold.
